# Pasteur and Hydrophobia

**Published:** 1891-01-03

**Authors:** 


### PASTEUR AND HYDROPHOBIA.

In the spring of the year Sir Henry Roscoe, at the Sunday Lecture Society, undertook to expound and defend Pasteur's inoculation treatment for hydrophobia. The expounder did not give an exposition, and the defender failed to defend. An able indictment of Pasteurism has been drawn up by Dr. Dolan, of Halifax, to which no competent reply is as yet forthcoming. Several writers in The Hospital have pointed out the *a priori* physiological difficulties which beset the method of Pasteur. No attempt of any importance has been made to meet these contentions. The position which this journal has taken up on the subject, and which it still holds, is this: That the *a priori* physiological difficulties in the way of Pasteur's antirabic treatment are exceedingly great; and that if the argument from analogy alone were held to be conclusive those difficulties would be insurmountable; but that, analogy notwithstanding, if undoubted facts and reliable statistics can be shown on the side of Pasteur, we shall bow to the actual and discard the *a priori* as misleading in the present instance. Pasteurism is still *sub judice*, and will probably long remain so. Those who value truth and a scientific reputation more than consistency will do well to recognise this fact.

